# Physical activity after unicompartmental knee arthroplasty: Long‐term participation, activity volume and patient‐reported limitations

**DOI:** 10.1002/jeo2.70929

**Published:** 2026-10-03

**Authors:** Waldo Gonzalez, Jorge Isla, Diego Barba, Daniela Erskine, Daniela Landea, Camila Tapia, David Figueroa

**Affiliations:** ^1^ Department of Traumatology, Knee and Arthroscopy Unit, Clinica Alemana Universidad del Desarrollo Santiago Chile; ^2^ Department of Traumatology, Knee and Arthroscopy Unit Hospital Regional de Coyhaique Aysen Chile; ^3^ Department of Traumatology, Knee and Arthroscopy Unit Hospital del Salvador Santiago Chile; ^4^ Department of Traumatology, Knee and Arthroscopy Unit Hospital Puerta de Hierro Andares Guadalajara Mexico; ^5^ Service of Physical Medicine and Rehabilitation, Clinica Alemana Santiago Chile

**Keywords:** activity limitations, patient‐reported outcomes, physical activity, return to sport, satisfaction, unicompartmental knee arthroplasty

## Abstract

**Purpose:**

To characterize long‐term patient‐reported sports and physical activity after unicompartmental knee arthroplasty (UKA), including participation, weekly volume, perceived exertion, time to resumption, limitations, satisfaction and sex‐based differences.

**Methods:**

This retrospective single‐institution study included 150 patients who underwent primary UKA between 2014 and 2022. An 11‐item electronic questionnaire administered in 2025 assessed activity at 5 years before UKA, 1 year before UKA, 1 year after UKA and at current follow‐up. Current participation was the primary outcome. Historical responses were treated as retrospectively recalled estimates. Mean follow‐up was 6.8 ± 2.4 years.

**Results:**

Of 190 contactable patients, 150 responded (78.9%). Mean age was 65.1 ± 10.9 years. Participation was recalled by 33.3% at 5 years before UKA, 8.0% at 1 year before UKA and 25.3% at 1 year after UKA; 33.3% reported current participation. Among current participants, 74.0% reported >3 h/week and 48.0% reported moderate‐or‐higher exertion. Overall, 49.3% resumed activity within 12 months, 42.0% perceived improvement and mean satisfaction was 3.84 ± 1.30. Activity‐related limitations and long‐term restrictions were reported by 75.3% and 30.0%, respectively. Most outcomes were similar between women and men; the fully adjusted association between male sex and improved intensity was not significant (adjusted odds ratio 2.08, 95% confidence interval 0.90–4.80).

**Conclusions:**

At long‐term follow‐up after UKA, one‐third of patients reported current sports or physical activity participation, and most current participants reported >3 h/week. However, only half resumed activity within 12 months, and residual limitations were common. Historical group‐level proportions should not be interpreted as individual return to activity or longitudinal recovery.

**Level of Evidence:**

Level IV.

**Abbreviations**
BMI: body mass index
CI: confidence interval
KOOS‐JR: Knee Injury and Osteoarthritis Outcome Score for Joint Replacement
OR: odds ratio
SD: standard deviation
TKA: total knee arthroplasty
UKA: unicompartmental knee arthroplasty

## INTRODUCTION

Unicompartmental knee arthroplasty (UKA) is an established treatment for appropriately selected patients with osteoarthritis confined to a single tibiofemoral compartment. In addition to pain relief and functional improvement, many patients expect to resume recreational sports and regular physical activity after surgery. Compared with total knee arthroplasty, UKA may facilitate postoperative activity through preservation of native knee structures, more physiological knee kinematics and faster early functional recovery in selected patients [5, 11, 13, 14, 21].

Previous studies have reported generally favourable rates of return to sport and physical activity after UKA [2, 18, 19, 23]. However, participation alone may provide an incomplete assessment of postoperative activity recovery. Patients may resume activity while reducing weekly volume, perceived intensity, activity demands or performance expectations. Residual symptoms, functional limitations, general health and patient‐perceived barriers may also influence long‐term activity participation [1, 4, 6, 7, 16, 22]. Nevertheless, studies simultaneously evaluating these complementary dimensions of activity after UKA remain limited.

The purpose of this study was to characterize long‐term patient‐reported sports and physical activity after UKA, with particular emphasis on whether participation was accompanied by meaningful activity volume and intensity. We hypothesized that current participation would be higher than during the year immediately preceding UKA, while residual activity‐related limitations would remain common.

## METHODS

### Study design and participants

This was a retrospective, single‐institution survey study evaluating long‐term patient‐reported sports and physical activity after primary UKA. Patients were identified from an institutional arthroplasty database. The study was approved by the Institutional Review Board of our institution (approval No. 2013‐01).

Potentially eligible patients were adults who underwent primary UKA between 2014 and 2022 and had demographic and surgical information available in the institutional database. Patients undergoing revision arthroplasty were excluded. Availability of a valid email address was considered a criterion for contactability rather than clinical eligibility. All contactable patients were invited to participate, and patients who completed the questionnaire constituted the final analytic cohort. Contactable nonrespondents were defined as invited patients who did not complete the survey.

No patient in the final analytic cohort had bilateral UKA. Therefore, the patient and the operated knee represented the same unit of analysis. Although the questionnaire used the terms ‘first joint replacement’ and ‘last joint replacement’, these references corresponded to the same index primary UKA for the present cohort. All preoperative and postoperative survey anchor timepoints were referenced to this index procedure.

### Data collection

Demographic and surgical variables were extracted from the institutional database, including age at surgery, sex, body mass index (BMI), date of surgery, operative side, compartment treated and postoperative complications. Time from surgery to survey completion was calculated using the exact date of the index UKA and the date on which the questionnaire was completed and was expressed in years.

Preoperative and postoperative knee‐specific functional outcomes were obtained from the institutional database using the Knee Injury and Osteoarthritis Outcome Score for Joint Replacement (KOOS‐JR) [15]. Preoperative and postoperative KOOS‐JR scores were summarized separately. Change in KOOS‐JR was calculated as the postoperative score minus the preoperative score and was analysed only among patients with paired assessments. The timing and number of available preoperative, postoperative and paired assessments are reported in the Results section. Analyses were based on available observations, and the denominator for each KOOS‐JR analysis was reported separately.

### Survey administration and outcome definitions

Eligible contactable patients received an electronic questionnaire through a secure survey platform. The survey was distributed in May 2025 and remained open until 1 August 2025. Nonresponders received up to three reminder emails during the survey period. Survey responses were linked to the institutional database using patient identifiers and were subsequently de‐identified for analysis.

An 11‐item questionnaire with branching logic was used to assess sports and recreational physical activity at four predefined anchor timepoints: 5 years before the index UKA, 1 year before the index UKA, 1 year after the index UKA and current follow‐up. The questionnaire additionally assessed weekly activity volume, perceived exertion, time to resumption of sports or physical activity, patient‐reported limitations, long‐term restrictions and their sources, perceived change in ability to participate and overall satisfaction with sports and physical activity participation (Data S1).

The primary study outcome was current participation in sports or physical activity. Secondary outcomes included retrospectively recalled participation at the three historical anchor timepoints, weekly activity volume among participating patients, perceived activity intensity, improvement in activity intensity, time to activity resumption, KOOS‐JR outcomes and item responses, patient‐reported limitations, long‐term restrictions, perceived performance and satisfaction. Sex‐based and age‐stratified analyses were considered secondary and exploratory.

The 5‐year preoperative anchor was selected a priori to provide a remote preoperative reference point against which activity during the year immediately preceding UKA could be contextualized. This anchor was not intended to represent a symptom‐free or objectively measured baseline. The questionnaire was not specifically validated for retrospective recall of sports participation, weekly activity volume or perceived exertion over intervals of this length. Accordingly, historical responses were treated as patient‐recalled estimates rather than objective longitudinal measurements.

At each anchor timepoint, sports or physical activity participation was defined according to the binary response to the question asking whether the patient participated in sports or physical activity at that specific timepoint.

Weekly activity volume was assessed separately using the predefined response categories of 0–3, 4–6, 7–9, 10–15 or more than 15 h per week. Patients reporting no participation at a given time point were classified separately as ‘no participation’ and were not included in the weekly activity‐volume distribution for that time point. Among patients reporting participation, the original 0–3 h/week response category was analysed as >0–3 h/week to distinguish low‐volume participation from complete inactivity. Weekly activity‐volume percentages were therefore calculated using the number of patients reporting participation at the corresponding timepoint as the denominator.

Perceived exertion was assessed at each anchor timepoint using categories adapted from the Borg Rating of Perceived Exertion Scale [3]: very light, light, moderate, vigorous, very hard and maximum effort. Current activity intensity was dichotomized as very light/light versus moderate‐or‐higher exertion. Moderate‐or‐higher exertion included moderate, vigorous, very hard and maximum effort responses.

Improvement in activity intensity was defined as a transition from very light or light exertion 1 year before the index UKA to moderate, vigorous, very hard or maximum effort 1 year after the index UKA. This outcome was analysed among patients with valid perceived‐exertion responses at both timepoints.

The perceived‐exertion items were administered to all respondents independently of their responses to the binary participation questions, and no branching logic was used to exclude nonparticipants from these items. Consequently, the derived intensity‐change outcome reflects a change in retrospectively recalled perceived‐exertion category across the full cohort and was not interpreted as an individual return‐to‐activity outcome.

Time to resumption of sports or physical activity after UKA was assessed using six predefined categories: less than 1 month, 1–3 months, 4–6 months, 7–12 months, more than 1 year or unable to return to sports or physical activity. For the main analysis, time to resumption was additionally dichotomized as 12 months or less versus more than 12 months or unable to return.

Patients reporting that they were unable to participate or participated for less time than desired were asked to identify their main limitation. Response options included joint pain, pain outside the knee, inability to perform required movements, medical advice, fear of damaging the joint, lack of confidence, general health problems, loss of interest in sport or physical activity or another reason. The presence of long‐term restrictions and the reported source of those restrictions were assessed separately.

Perceived change in the ability to participate in sports or physical activity compared with before surgery was assessed using a 5‐point Likert‐type scale ranging from significantly declined to significantly improved. Satisfaction with the ability to participate after UKA was assessed using a 5‐point Likert‐type scale ranging from very unsatisfied to very satisfied [10].

Sports and recreational activities included in the questionnaire were classified according to previously published activity‐impact classifications and consensus recommendations from arthroplasty societies [8, 9, 12].

### Statistical analysis

Categorical variables were summarized as counts and percentages. Continuous variables were summarized as means and standard deviations. Because time from surgery to survey completion could have a non‐normal distribution, it was additionally summarized using the median, interquartile range (IQR) and range.

The survey response rate was calculated as the number of completed questionnaires divided by the number of contactable patients invited to participate. The proportion of potentially eligible patients included in the final cohort was reported separately.

Current sports and physical activity participation, the primary outcome, was summarized as a count and percentage. Participation at the three historical anchor timepoints was also summarized descriptively. Because identical cohort‐level proportions at different timepoints do not establish that the same patients participated at both timepoints, these results were interpreted as retrospectively recalled participation proportions rather than individual return‐to‐activity rates.

Weekly activity volume was analysed among patients reporting participation at each corresponding timepoint. Patients reporting no participation were presented separately and were not included in the denominator for the weekly activity‐volume distribution. The proportion of participating patients reporting more than 3 h of activity per week was additionally calculated at each timepoint. Comparisons across the four anchor timepoints were descriptive and were not interpreted as prospectively measured longitudinal changes.

Sex‐based comparisons were performed for demographic and surgical characteristics, KOOS‐JR outcomes, current participation, weekly activity volume, current perceived exertion, improvement in activity intensity, time to activity resumption, functional symptoms, activity‐related limitations, perceived performance and satisfaction. These comparisons were considered exploratory.

Categorical variables were compared using the *χ* ^2^ test or Fisher's exact test, as appropriate. Continuous variables were compared using the Student's *t* test for normally distributed variables and the Mann–Whitney *U* test for non‐normally distributed variables. The statistical test and number of observations used for each KOOS‐JR comparison are reported with the corresponding results.

The association between sex and improvement in activity intensity was evaluated using logistic regression. The unadjusted model included sex as the independent variable. The primary adjusted model included sex and age. A sensitivity model additionally adjusted for BMI, time from surgery to survey completion and compartment treated. Odds ratios (ORs), adjusted odds ratios (aORs) and 95% confidence intervals (CIs) were reported.

Exploratory age‐stratified analyses were performed for patients younger than 60 years and those aged 60 years or older. A sex‐by‐age interaction term was evaluated in a logistic regression model adjusted for BMI.

No a priori sample‐size or power calculation was performed because all potentially eligible patients treated during the study period were screened and all contactable patients were invited to participate. No adjustment for multiple comparisons was applied because the sex‐based and subgroup analyses were exploratory. Missing values were not imputed, and analyses were performed using the available data for each outcome.

All statistical tests were two‐sided, and statistical significance was set at *p* < 0.05. All analyses were performed using Stata version 19.0.

## RESULTS

### Patient flow and characteristics

A total of 213 patients who underwent primary UKA between 2014 and 2022 were potentially eligible for inclusion. Of these, 23 did not have valid electronic contact information and were considered noncontactable. The remaining 190 patients were invited to participate in the electronic survey. A total of 150 patients completed the questionnaire and constituted the final analytic cohort, whereas 40 invited patients did not respond. The response rate was 78.9% among invited patients, and the final cohort represented 70.4% of the potentially eligible population.

The final cohort included 70 women (46.7%) and 80 men (53.3%). Mean age at surgery was 65.1 ± 10.9 years, and mean BMI was 28.5 ± 4.3 kg/m^2^. Most procedures involved the medial compartment (101/150, 67.3%), and 82 UKAs (54.7%) were performed on the right knee.

### Postoperative complications

Twelve postoperative complications were recorded (8.0%). Arthrofibrosis was the most frequent complication, occurring in 3 patients (25.0% of complications), followed by deep vein thrombosis in 2 (16.7%) and cellulitis in 2 (16.7%). Other complications included prosthetic loosening in 1 patient (8.3%), an intra‐articular loose body in 1 (8.3%), postoperative bleeding with a decrease in haematocrit in 1 (8.3%), an infected seroma in 1 (8.3%) and a periprosthetic fracture in 1 (8.3%).

Two patients underwent reoperation for arthrofibrosis. No implant revision procedures were performed, and no additional complication‐related hospital readmissions were identified.

At current follow‐up, 7 of the 12 patients with a postoperative complication (58.3%) reported current sports or physical activity participation, compared with 43 of the 138 patients without a recorded complication (31.2%). Given the small number and clinical heterogeneity of complications, these findings were considered descriptive.

Women and men were similar in age (65.5 ± 11.5 vs. 64.8 ± 10.3 years; *p* = 0.68) and BMI (28.7 ± 4.0 vs. 28.4 ± 4.5 kg/m^2^; *p* = 0.68). Medial UKA was more frequent among women than men (78.6% vs. 57.5%; *p* = 0.006). No statistically significant sex‐based differences were observed for operative side (right knee, 62.9% vs. 47.5%; *p* = 0.06) or postoperative complications (8.6% vs. 7.5%; *p* = 0.80).

Time from surgery to survey completion was 6.8 ± 2.4 years, with a median of 6.7 years (IQR, 4.8–8.9 years) and a range of 2.5–11.4 years. Mean follow‐up was 6.3 ± 2.4 years in women and 7.3 ± 2.3 years in men (*p* = 0.01). Demographic and surgical characteristics are summarized in Table 1.

**Table 1:** Demographic and surgical characteristics by sex.

Characteristic	Overall (*N* = 150)	Women (*n* = 70)	Men (*n* = 80)	*p* value
Age, years, mean (SD)	65.1 (10.9)	65.5 (11.5)	64.8 (10.3)	0.68
BMI, kg/m^2^, mean (SD)	28.5 (4.3)	28.7 (4.0)	28.4 (4.5)	0.68
Surgical characteristics				
Time from surgery to survey, years, mean (SD)	6.8 (2.4)	6.3 (2.4)	7.3 (2.3)	**0.01**
Medial UKA	101 (67.3)	55 (78.6)	46 (57.5)	**0.006**
Right knee	82 (54.7)	44 (62.9)	38 (47.5)	**0.06**
Postoperative complication	12 (8.0)	6 (8.6)	6 (7.5)	0.80

*Note*: Values are presented as *n* (%) unless otherwise indicated.

Abbreviations: BMI, body mass index; SD, standard deviation; UKA, unicompartmental knee arthroplasty.

### KOOS‐JR outcomes

Preoperative and postoperative KOOS‐JR scores are summarized in Table 2. Preoperative KOOS‐JR assessments were available for 26 patients, including 12 women and 14 men, and were obtained a median of 31 days before surgery (IQR, 18–44 days). Postoperative assessments were available for all 150 patients and were obtained at a median of 18.2 months after UKA (IQR, 17.0–19.6 months). Paired preoperative and postoperative assessments were therefore available for 26 patients.

**Table 2:** Preoperative, postoperative and change in KOOS‐JR scores overall and by sex.

KOOS‐JR outcome	Overall	Women	Men	*p* value
Preoperative KOOS‐JR	43.1 (9.6), *n* = 26	46.3 (10.8), *n* = 12	40.4 (7.8), *n* = 14	0.12
Postoperative KOOS‐JR	70.3 (14.7), *n* = 150	79.4 (11.6), *n* = 70	62.4 (12.4), *n* = 80	<0.001
Pre‐to‐postoperative change	27.1 (13.5), *n* = 26	33.0 (16.3), *n* = 12	22.0 (8.2), *n* = 14	0.06

*Note*: Values are presented as mean (SD). Change was calculated as the postoperative score minus the preoperative score among patients with paired assessments.

Abbreviations: KOOS‐JR, Knee Injury and Osteoarthritis Outcome Score for Joint Replacement; SD, standard deviation.

For patients with available preoperative assessments, the overall mean preoperative KOOS‐JR score was 43.1 ± 9.6 points. The overall mean postoperative KOOS‐JR score was 70.3 ± 14.7 points, and the mean pre‐to‐postoperative improvement among patients with paired assessments was 27.1 ± 13.5 points.

Preoperative KOOS‐JR scores did not differ significantly between women and men (46.3 ± 10.8 vs. 40.4 ± 7.8; Mann–Whitney *U* test; *p* = 0.12). Postoperative KOOS‐JR scores were significantly higher in women than in men (79.4 ± 11.6 vs. 62.4 ± 12.4; Student's *t* test; *p* < 0.001). Among patients with paired assessments, pre‐to‐postoperative improvement was numerically greater in women than men, although the difference did not reach statistical significance (33.0 ± 16.3 vs. 22.0 ± 8.2 points; Mann–Whitney *U* test; *p* = 0.06). Preoperative, postoperative and change scores are summarized in Table 2.

### Sports and physical activity participation

Sports or physical activity participation was retrospectively reported by 50 patients (33.3%) 5 years before UKA and by 12 patients (8.0%) 1 year before UKA. At 1 year after UKA, 38 patients (25.3%) reported participation, while 50 patients (33.3%) reported participating at current follow‐up.

The proportion reporting current participation, which represented the primary study outcome, was therefore 33.3%. Current participation was reported by 23 of 70 women (32.9%) and 27 of 80 men (33.8%), with no statistically significant sex‐based difference (*p* = 0.91).

Historical values represent retrospectively recalled group‐level participation proportions and do not establish patient‐level transitions or individual return‐to‐activity rates (Figure 1).

**Figure 1:**
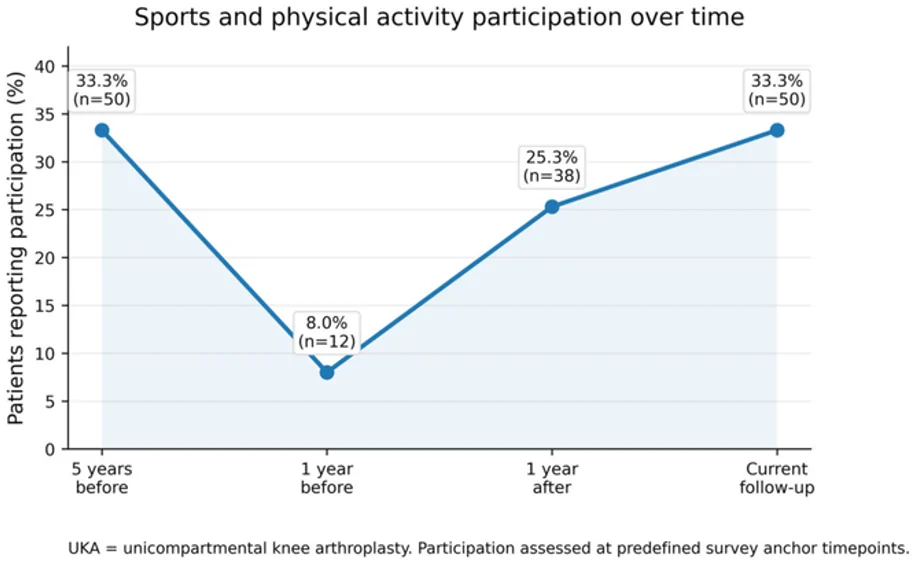
Retrospectively recalled sports and physical activity participation across survey timepoints. Participation was assessed at four predefined anchor timepoints: 5 years before UKA, 1 year before UKA, 1 year after UKA and current follow‐up. Values represent the proportion of respondents reporting participation at each timepoint. These group‐level proportions should not be interpreted as patient‐level transitions or individual return‐to‐activity rates. UKA, unicompartmental knee arthroplasty.

### Weekly activity volume and exertion levels

Weekly activity volume was analysed among patients reporting sports or physical activity participation at each corresponding time point. Patients reporting no participation were classified separately and were not included in the activity‐volume distributions.

Five years before UKA, among the 50 patients reporting participation, 20 (40.0%) reported >0–3 h/week, 22 (44.0%) reported 4–6 h/week and 8 (16.0%) reported 10–15 h/week. No participants reported 7–9 or more than 15 h/week. Overall, 30 of 50 participants (60.0%) reported more than 3 h/week.

One year before UKA, among the 12 participating patients, 6 (50.0%) reported >0–3 h/week and 6 (50.0%) reported 4–6 h/week. Thus, 6 of 12 participants (50.0%) reported more than 3 h/week.

One year after UKA, among the 38 participating patients, 25 (65.8%) reported >0–3 h/week and 13 (34.2%) reported 4–6 h/week. Therefore, 13 of 38 participants (34.2%) reported more than 3 h/week.

At current follow‐up, among the 50 participating patients, 13 (26.0%) reported >0–3 h/week, 12 (24.0%) reported 4–6 h/week, 13 (26.0%) reported 7–9 h/week and 12 (24.0%) reported 10–15 h/week. No participant reported more than 15 h/week. Overall, 37 of 50 current participants (74.0%) reported more than 3 h of sports or physical activity per week (Table 3).

**Table 3:** Weekly sports and physical activity volume among participating patients.

Weekly activity volume	5 years before UKA *N* = 50	1 year before UKA *N* = 12	1 year after UKA *N* = 38	Current *N* = 50
>0–3 h/week	20 (40.0)	6 (50.0)	25 (65.8)	13 (26.0)
4–6 h/week	22 (44.0)	6 (50.0)	13 (34.2)	12 (24.0)
7–9 h/week	0	0	0	13 (26.0)
10–15 h/week	8 (16.0)	0	0	12 (24.0)
>15 h/week	0	0	0	0

*Note*: Values are presented as *n* (%). Percentages were calculated using the number of patients reporting sports or physical activity participation at each corresponding time point as the denominator. Patients reporting no participation were analysed separately and were not included in the 0–3 h/week category.

Abbreviation: UKA, unicompartmental knee arthroplasty.

### Perceived activity intensity

Among the 50 patients reporting current participation, perceived exertion was rated as very light by 5 patients (10.0%), light by 21 (42.0%), moderate by 16 (32.0%), vigorous by 6 (12.0%) and very hard by 2 (4.0%). No patient reported maximum effort. Thus, 24 of 50 current participants (48.0%) reported moderate‐or‐higher exertion.

Moderate‐or‐higher current exertion was reported by 11 of 23 participating women (47.8%) and 13 of 27 participating men (48.1%), with no significant sex‐based difference (*p* = 0.98).

All 150 respondents completed the perceived‐exertion items at both 1 year before and 1 year after UKA because these items were administered independently of the binary participation questions. Improvement in activity intensity, defined as a transition from very light or light exertion 1 year before UKA to moderate‐or‐higher exertion 1 year after UKA, was observed in 37 patients (24.7%). Improvement was observed in 12 women (17.1%) and 25 men (31.3%; *p* = 0.04).

### Time to resumption of sports or physical activity

Time to resumption of sports or physical activity after UKA was reported as less than 1 month by 8 patients (5.3%), 1–3 months by 20 (13.3%), 4–6 months by 28 (18.7%), 7–12 months by 18 (12.0%) and more than 1 year by 32 (21.3%). A further 44 patients (29.3%) reported that they were unable to resume sports or physical activity.

Overall, 74 patients (49.3%) reported resuming sports or physical activity within 12 months after UKA. Resumption within 12 months was reported by 32 women (45.7%) and 42 men (52.5%), with no statistically significant sex‐based difference (*p* = 0.40).

### Postoperative KOOS‐JR item responses and functional symptoms

Moderate‐to‐extreme morning stiffness was reported by 38 patients (25.3%), without a significant difference between women and men (22.9% vs. 27.5%; *p* = 0.51). Moderate‐to‐extreme pain with twisting or pivoting and with full knee extension was reported by 12 patients (8.0%) for each item. Moderate‐to‐extreme pain during stair ascent or descent was reported by 26 patients (17.3%), while 12 patients (8.0%) reported moderate‐to‐extreme pain while standing upright.

Moderate‐to‐extreme difficulty rising from a seated position was reported by 24 patients (16.0%), with a nonsignificant trend toward a higher frequency among men than women (21.3% vs. 10.0%; *p* = 0.06). Moderate‐to‐extreme difficulty bending to the floor or picking up an object was reported by 50 patients (33.3%).

### Perceived performance and satisfaction

When asked to compare their current ability to participate in sports or physical activity with their preoperative ability, 20 patients (13.3%) reported a significant decline, 24 (16.0%) reported a moderate decline, 43 (28.7%) reported approximately the same ability, 39 (26.0%) reported moderate improvement and 24 (16.0%) reported significant improvement.

Overall, 63 patients (42.0%) reported either moderate or significant improvement. Improvement was reported by 26 women (37.1%) and 37 men (46.3%), without a statistically significant sex‐based difference (*p* = 0.26).

Satisfaction with the ability to participate in sports or physical activity was rated as very unsatisfied by 11 patients (7.3%), unsatisfied by 15 (10.0%), neither satisfied nor unsatisfied by 28 (18.7%), satisfied by 29 (19.3%) and very satisfied by 67 (44.7%).

Mean satisfaction was 3.84 ± 1.30 points on the 5‐point scale. Mean satisfaction was 3.86 ± 1.30 among women and 3.83 ± 1.30 among men, corresponding to rounded values of 3.9 and 3.8, respectively. No statistically significant sex‐based difference was observed (*p* = 0.88).

### Patient‐reported limitations and long‐term restrictions

Overall, 113 patients (75.3%) reported at least one limitation affecting sports or physical activity. Limitations were reported by 49 women (70.0%) and 64 men (80.0%), without a statistically significant difference (*p* = 0.15).

Among the 113 patients reporting limitations, inability to perform required movements was identified by 36 patients (31.9%), joint pain by 27 (23.9%) and general health problems by 26 (23.0%). Loss of interest in sports or physical activity and another reason were each reported by 12 patients (10.6%). Pain outside the knee, medical advice, fear of damaging the prosthesis and lack of confidence were not selected as the primary limitation.

Long‐term restrictions on sports or physical activity were reported by 45 patients (30.0%), including 18 women (25.7%) and 27 men (33.8%); this difference was not statistically significant (*p* = 0.28).

Among the 45 patients reporting long‐term restrictions, the source was identified as self‐imposed by 20 patients (44.4%), the treating surgeon by 9 (20.0%), a physical or occupational therapist by 6 (13.3%), another physician by 4 (8.9%), a health or fitness professional by 2 (4.4%), friends or family by 2 (4.4%) and online information by 2 (4.4%).

### Sex‐based analyses

Most participation, activity‐volume, current perceived‐intensity, time‐to‐resumption, functional‐symptom, limitation, perceived‐performance and satisfaction outcomes were similar between women and men. The principal activity‐related sex difference was observed for improvement in perceived activity intensity.

In the unadjusted logistic regression model, men had higher odds of improving activity intensity than women (OR 2.20, 95% CI 1.01–4.80). After adjustment for age, the association remained statistically significant (adjusted OR 2.22, 95% CI 1.01–4.86).

In the sensitivity model, additionally adjusting for BMI, time from surgery to survey completion and compartment treated, the magnitude of the association remained similar but no longer reached conventional statistical significance (adjusted OR 2.08, 95% CI 0.90–4.80; *p* = 0.08).

In exploratory age‐stratified analyses, the sex difference in improvement in activity intensity was borderline among patients younger than 60 years (*p* = 0.05) and was not statistically significant among patients aged 60 years or older (*p* = 0.28). The sex‐by‐age interaction term adjusted for BMI was not statistically significant (*p* = 0.36).

## DISCUSSION

The principal findings of this study were that 33.3% of patients reported current participation in sports or physical activity and, among these participants, 74.0% performed more than 3 h of activity per week and 48.0% reported moderate‐or‐higher exertion. Approximately half of the overall cohort resumed activity within 12 months, whereas activity‐related limitations and long‐term restrictions were reported by 75.3% and 30.0% of patients, respectively. Most activity‐related outcomes were similar between women and men, and the association between male sex and improvement in activity intensity was attenuated after comprehensive adjustment.

Participation and activity volume provided complementary information. Although only one‐third of the cohort reported current participation, nearly three‐quarters of these active patients performed more than 3 h per week. Therefore, cohort‐level participation should not be interpreted as a direct measure of activity exposure among those who remain active. Separating nonparticipants from patients reporting >0–3 h/week was important because combining these groups would have underestimated the activity volume of participating patients.

The retrospectively recalled participation proportions were 33.3% 5 years before UKA, 8.0% 1 year before UKA, 25.3% 1 year after UKA and 33.3% at current follow‐up. These results suggest that patients perceived lower participation during the year immediately preceding surgery. However, identical proportions at 5 years before UKA and current follow‐up do not demonstrate that the same patients participated at both time points. Given the prolonged recall interval, these findings should be interpreted as group‐level patient‐reported patterns rather than evidence of individual return to activity or longitudinal recovery.

The current participation proportion was lower than return‐to‐sport rates reported in previous studies and systematic reviews, which have generally ranged from approximately 74% to 100% after UKA [2, 16, 18, 19, 23, 24]. More recent studies have also reported favourable activity outcomes in selected active patients after UKA [4, 17, 18]. These results are not directly comparable with the present study because many previous investigations calculated return to sport only among patients who were active before surgery, whereas our current participation proportion used the entire cohort as the denominator. Differences in patient selection, activity definitions, follow‐up duration, rehabilitation and recreational practices may also explain the variation across studies [2, 4, 16, 19, 23].

Activity volume and perceived intensity should also be considered separately. Among current participants, 74.0% reported more than 3 h of activity per week, but only 48.0% described their exertion as moderate or higher. Previous literature has shown that patients may modify the type, intensity or demands of activity after knee arthroplasty and often transition toward lower‐impact modalities [2, 9, 16, 17, 19, 23]. Therefore, participation, volume and exertion represent distinct dimensions of postoperative activity.

Time to resumption, perceived performance and satisfaction provided additional context. Only 49.3% resumed sports or physical activity within 12 months, while 21.3% required more than 1 year and 29.3% reported being unable to resume activity. Nevertheless, 42.0% perceived their current ability as improved, and mean satisfaction was 3.84 on a 5‐point scale. Patients may therefore perceive meaningful benefit and remain satisfied without returning rapidly, resuming the same activity or achieving a specific activity level [4, 6, 18, 19].

Residual symptoms and limitations remained frequent. Three‐quarters of respondents reported at least one activity‐related limitation, most commonly inability to perform required movements, joint pain and general health problems. Long‐term restrictions were reported by 30.0%, and the most frequent source was self‐imposed restriction. Previous studies have similarly identified symptoms, comorbidities, functional capacity, patient perceptions and professional recommendations as potential determinants of postoperative sports participation [1, 4, 6, 7, 17, 20]. These findings suggest that postoperative counselling should address not only whether activity is permitted, but also expected timing, intensity, functional demands and safe progression. Contemporary recommendations generally support low‐impact activities and selected intermediate‐impact activities, with individualized decision‐making for higher‐impact sports [1, 8, 9, 17, 20].

Most activity‐related outcomes were comparable between women and men. Although men had higher odds of improving activity intensity in the unadjusted and age‐adjusted models, this association was no longer statistically significant after adjustment for BMI, follow‐up duration and compartment treated. Because these analyses were exploratory and involved multiple comparisons, the finding should not be interpreted as evidence of a biological sex difference. Postoperative KOOS‐JR scores were higher among women, while participation and activity outcomes were similar by sex. This supports the concept that knee‐specific functional scores and real‐world activity behaviour are related but not interchangeable [15].

Overall, these findings support a multidimensional evaluation of physical activity after UKA. Participation alone does not describe weekly volume, perceived intensity, time to resumption, satisfaction or residual barriers. Future prospective studies should preserve patient‐level transitions across timepoints, distinguish inactivity from low‐volume participation and incorporate objective activity monitoring to provide a more precise assessment of postoperative activity.

### Limitations

This study has several limitations. First, all activity data were collected through a single survey administered in 2025, and participation, weekly activity volume and perceived exertion at the historical timepoints were assessed retrospectively. For some patients, the 5‐year preoperative anchor required recalling activity performed more than a decade earlier, and the questionnaire was not validated for recall over intervals of this length. Historical results should therefore be interpreted as approximate patient‐reported patterns rather than objective longitudinal measurements.

Second, activity outcomes were self‐reported and were not confirmed using wearable devices, objective activity monitoring or performance‐based testing. Although nonparticipants were analysed separately from patients reporting >0–3 h/week, the lowest activity‐volume category remained broad. The survey also did not establish whether patients resumed the same sport, impact level, weekly frequency, competitive level or performance level as before surgery.

Third, cohort‐level participation proportions at different timepoints do not establish individual return to activity. In addition, follow‐up varied from 2.5 to 11.4 years, and current activity may have been influenced by aging, comorbidities, lifestyle changes and factors unrelated to the operated knee. The inclusion of only electronically contactable respondents may also have introduced selection and nonresponse bias.

Finally, preoperative KOOS‐JR assessments were available for only 26 patients. No a priori sample‐size calculation was performed, and the sex‐based and age‐stratified analyses were exploratory and involved multiple comparisons. As this was a retrospective, single‐institution study, the findings may not be generalizable and should be interpreted as descriptive rather than definitive evidence of longitudinal activity recovery after UKA.

## CONCLUSION

At a mean follow‐up of 6.8 years after UKA, 33.3% of patients reported current sports or physical activity participation. Among current participants, 74.0% reported more than 3 h of activity per week and 48.0% reported moderate‐or‐higher exertion. However, only 49.3% of the overall cohort resumed activity within 12 months, while activity‐related limitations and long‐term restrictions remained common. Because historical activity was assessed retrospectively and summarized using group‐level proportions, these findings should not be interpreted as evidence of individual return to activity or longitudinal recovery.

## AUTHOR CONTRIBUTIONS

All the authors contributed to the design, analyses and reporting for this manuscript. Both authors read and approved the final submitted manuscript.

## CONFLICT OF INTEREST STATEMENT

The authors declare no conflicts of interest.

## ETHICS STATEMENT

The study was approved by the Institutional Review Board of Clinica Alemana Santiago (approval no. 2013‐01). Informed consent was obtained from all patients for the use of their data.

## Supporting information

Supporting File

## Data Availability

Data are available on request from the authors.
