# Supplementary material for: Physical activity after unicompartmental knee arthroplasty: Long‐term participation, activity volume and patient‐reported limitations
Source: J Exp Orthop. 2026 Oct 3;13(4):e70929. doi: 10.1002/jeo2.70929 (PMC13633556; doi:10.1002/jeo2.70929)
Supplement: Supplementary file 1 [file JEO2-13-e70929-s001.docx]

**Appendix 1. Study Survey**

1. Which of the following joint replacement(s) have you had?

a. Right Unicompartmental Knee Replacement (Partial Knee Replacement)

b. Left Unicompartmental Knee Replacement (Partial Knee Replacement)

2. Did you participate in sports or physical activities around the following times?

a. 5 years before your first joint replacement

b. 1 year before your first joint replacement

c. 1 year after your last joint replacement

d. Currently

3. How many hours per week did you participate in sports or physical activity around the following times?

a. 5 years before your first joint replacement

i. 0-3 hours per week

ii. 4-6 hours per week

iii. 7-9 hours per week

iv. 10-15 hours per week

v. More than 15 hours per week

b. 1 year before your first joint replacement

i. 0-3 hours per week

ii. 4-6 hours per week

iii. 7-9 hours per week

iv. 10-15 hours per week

v. More than 15 hours per week

c. 1 year after your last joint replacement

i. 0-3 hours per week

ii. 4-6 hours per week

iii. 7-9 hours per week

iv. 10-15 hours per week

v. More than 15 hours per week

d. Currently

i. 0-3 hours per week

ii. 4-6 hours per week

iii. 7-9 hours per week

iv. 10-15 hours per week

v. More than 15 hours per week

4. What sports or physical activities did you participate in around the following times?

a. Badminton

i. 5 years before your first joint replacement

ii. 1 year before your first joint replacement

iii. 1 year after your last joint replacement

iv. Currently

b. Basketball

i. 5 years before your first joint replacement

ii. 1 year before your first joint replacement

iii. 1 year after your last joint replacement

iv. Currently

c. Boxing

i. 5 years before your first joint replacement

ii. 1 year before your first joint replacement

iii. 1 year after your last joint replacement

iv. Currently

d. Cycling

i. 5 years before your first joint replacement

ii. 1 year before your first joint replacement

iii. 1 year after your last joint replacement

iv. Currently

e. Extreme Sports

i. 5 years before your first joint replacement

ii. 1 year before your first joint replacement

iii. 1 year after your last joint replacement

iv. Currently

f. Golf

i. 5 years before your first joint replacement

ii. 1 year before your first joint replacement

iii. 1 year after your last joint replacement

iv. Currently

g. Gymnastics

i. 5 years before your first joint replacement

ii. 1 year before your first joint replacement

iii. 1 year after your last joint replacement

iv. Currently

h. Jogging

i. 5 years before your first joint replacement

ii. 1 year before your first joint replacement

iii. 1 year after your last joint replacement

iv. Currently

i. Martial Arts (ex. Karate, Kung fu, Taekwondo, Jiu-Jitsu, etc.)

i. 5 years before your first joint replacement

ii. 1 year before your first joint replacement

iii. 1 year after your last joint replacement

iv. Currently

j. Motorsport

i. 5 years before your first joint replacement

ii. 1 year before your first joint replacement

iii. 1 year after your last joint replacement

iv. Currently

k. Mountain Biking

i. 5 years before your first joint replacement

ii. 1 year before your first joint replacement

iii. 1 year after your last joint replacement

iv. Currently

l. Rugby

i. 5 years before your first joint replacement

ii. 1 year before your first joint replacement

iii. 1 year after your last joint replacement

iv. Currently

m. Running

i. 5 years before your first joint replacement

ii. 1 year before your first joint replacement

iii. 1 year after your last joint replacement

iv. Currently

n. Skiing

i. 5 years before your first joint replacement

ii. 1 year before your first joint replacement

iii. 1 year after your last joint replacement

iv. Currently

o. Snowboarding

i. 5 years before your first joint replacement

ii. 1 year before your first joint replacement

iii. 1 year after your last joint replacement

iv. Currently

p. Soccer

i. 5 years before your first joint replacement

ii. 1 year before your first joint replacement

iii. 1 year after your last joint replacement

iv. Currently

q. Padel

i. 5 years before your first joint replacement

ii. 1 year before your first joint replacement

iii. 1 year after your last joint replacement

iv. Currently

r. Swimming

i. 5 years before your first joint replacement

ii. 1 year before your first joint replacement

iii. 1 year after your last joint replacement

iv. Currently

s. Tennis

i. 5 years before your first joint replacement

ii. 1 year before your first joint replacement

iii. 1 year after your last joint replacement

iv. Currently

t. Volleyball

i. 5 years before your first joint replacement

ii. 1 year before your first joint replacement

iii. 1 year after your last joint replacement

iv. Currently

u. Walking (eg, Recreational, not including activities of daily life)

i. 5 years before your first joint replacement

ii. 1 year before your first joint replacement

iii. 1 year after your last joint replacement

iv. Currently

5. How would you rate the intensity of your sports/physical activities around the following times?

a. 5 years before your first joint replacement

i. Very light - hardly any exertion, but more than sleeping, watching TV, etc.

ii. Light - you could keep going for hours, easy to breathe and have conversation

iii. Moderate - you're breathing heavily but you can have a conversation

iv. Vigorous - you're short of breath. You can speak, but only about one sentence at a time

v. Very hard - you can barely breathe, and can only say a few words at a time

vi. Maximum effort - you're completely out of breath and can't speak

b. 1 year before your first joint replacement

i. Very light - hardly any exertion, but more than sleeping, watching TV, etc.

ii. Light - you could keep going for hours, easy to breathe and have conversation

iii. Moderate - you're breathing heavily but you can have a conversation

iv. Vigorous - you're short of breath. You can speak, but only about one sentence at a time

v. Very hard - you can barely breathe, and can only say a few words at a time

vi. Maximum effort - you're completely out of breath and can't speak

c. 1 year after your last joint replacement

i. Very light - hardly any exertion, but more than sleeping, watching TV, etc.

ii. Light - you could keep going for hours, easy to breathe and have conversation

iii. Moderate - you're breathing heavily but you can have a conversation

iv. Vigorous - you're short of breath. You can speak, but only about one sentence at a time

v. Very hard - you can barely breathe, and can only say a few words at a time

vi. Maximum effort - you're completely out of breath and can't speak

d. Currently

i. Very light - hardly any exertion, but more than sleeping, watching TV, etc.

ii. Light - you could keep going for hours, easy to breathe and have conversation

iii. Moderate - you're breathing heavily but you can have a conversation

iv. Vigorous - you're short of breath. You can speak, but only about one sentence at a time

v. Very hard - you can barely breathe, and can only say a few words at a time

vi. Maximum effort - you're completely out of breath and can't speak

6. After your last joint replacement surgery, how much time did it take to resume sports or physical activities?

a. Less than 1 month

b. 1-3 months

c. 4-6 months

d. 7-12 months

e. More than 1 year

f. Unable to return to sports/physical activity

7. After having a joint replacement, if you are unable to participate in sports/physical activities, or if you participate in them for a shorter amount of time than you would like, what are your main limitations?

a. Not applicable (no limitations)

b. Joint pain

c. Pain other than in my knee

d. Unable to do required movements

e. Medical advice

f. Fear of damaging joint

g. Lack of confidence

h. General health problems

i. Loss of interest in sport/activity

j. Other

8. Do you have any long-term restrictions on participating in sports/physical activities?

a. Yes

b. No

9. Who or what is the source of your restrictions on participating in sports/physical activities? (Note: short-term restrictions within the first few months after surgery do not count)

a. Surgeon

b. Another physician

c. Physical/occupational therapist

d. Health/ fitness coach or instructor

e. Friends or family

f. Online reading

g. Self

10. Compared to before having joint replacement surgery, how would you rate your current participation in sports or physical activities?

a. Significantly declined: I am able to participate in sports/physical activities significantly less or worse than prior to surgery

b. Moderately decline: I am able to participate in sports/physical activities somewhat less or worse than prior to surgery

c. About the same: I am able to participate in sports/physical activities at or about the same level as prior to surgery

d. Moderately improved: I am able to participate in sports/physical activities somewhat more or better than prior to surgery

e. Significantly improved: I am able to participate in sports/physical activities significantly more or better than prior to surgery

11. After having joint replacement surgery, on a scale of 1-5, how satisfied are you with your ability to participate in sports or physical

activities?

a. Very unsatisfied

b. Unsatisfied

c. Neither satisfied or unsatisfied

d. Satisfied

e. Very satisfied.
